# Stroke-induced changes to immune function and their relevance to increased risk of severe COVID-19 disease

**DOI:** 10.1093/discim/kyac004

**Published:** 2022-08-11

**Authors:** Laura McCulloch, Isobel C Mouat, Kieron South, Barry W McColl, Stuart M Allan, Craig J Smith

**Affiliations:** Centre for Inflammation Research, University of Edinburgh, Edinburgh, UK; Centre for Inflammation Research, University of Edinburgh, Edinburgh, UK; Division of Neuroscience and Experimental Psychology, School of Biological Sciences, Faculty of Biology, Medicine and Health, University of Manchester, Manchester, UK; Lydia Becker Institute of Immunology and Inflammation, Division of Immunology, Immunity to Infection and Respiratory Medicine, School of Biological Sciences, Faculty of Biology, Medicine and Health, University of Manchester, Manchester Academic Health Science Centre, Manchester, UK; Geoffrey Jefferson Brain Research Centre, Manchester Academic Health Science Centre, Northern Care Alliance NHS Foundation Trust, University of Manchester, Manchester, UK; UK Dementia Research Institute, University of Edinburgh, Edinburgh, UK; Division of Neuroscience and Experimental Psychology, School of Biological Sciences, Faculty of Biology, Medicine and Health, University of Manchester, Manchester, UK; Lydia Becker Institute of Immunology and Inflammation, Division of Immunology, Immunity to Infection and Respiratory Medicine, School of Biological Sciences, Faculty of Biology, Medicine and Health, University of Manchester, Manchester Academic Health Science Centre, Manchester, UK; Geoffrey Jefferson Brain Research Centre, Manchester Academic Health Science Centre, Northern Care Alliance NHS Foundation Trust, University of Manchester, Manchester, UK; Lydia Becker Institute of Immunology and Inflammation, Division of Immunology, Immunity to Infection and Respiratory Medicine, School of Biological Sciences, Faculty of Biology, Medicine and Health, University of Manchester, Manchester Academic Health Science Centre, Manchester, UK; Geoffrey Jefferson Brain Research Centre, Manchester Academic Health Science Centre, Northern Care Alliance NHS Foundation Trust, University of Manchester, Manchester, UK; Greater Manchester Comprehensive Stroke Centre, Manchester Centre for Clinical Neurosciences, Manchester Academic Health Science Centre, Salford Royal NHS Foundation Trust, Salford, UK

## Abstract

As the COVID-19 pandemic moves towards endemic disease, it remains of key importance to identify groups of individuals vulnerable to severe infection and understand the biological factors that mediate this risk. Stroke patients are at increased risk of developing severe COVID-19, likely due to stroke-induced alterations to systemic immune function. Furthermore, immune responses associated with severe COVID-19 in patients without a history of stroke parallel many of the immune alterations induced by stroke, possibly resulting in a compounding effect that contributes to worsened disease severity. In this review, we discuss the changes to systemic immune function that likely contribute to augmented COVID-19 severity in patients with a history of stroke and the effects of COVID-19 on the immune system that may exacerbate these effects.

## Introduction

Coronavirus disease 2019 (COVID-19) was declared a pandemic by the World Health Organisation on 11 March 2020. The virus responsible for COVID-19 was identified as a novel coronavirus, Severe Acute Respiratory Syndrome Coronavirus 2 (SARS-CoV-2), which uses the angiotensin converting enzyme 2 receptor to enter and infect cells [1]. Manifestation of COVID-19 ranges from asymptomatic to severe disease, with symptoms including fever, dry cough, shortness of breath, and fatigue, and can also include headache, muscle and joint pain, nausea and diarrhoea, dysregulated blood clotting, loss of smell and taste, and acute cardiac and renal injury [2–8]. Patients can also experience breathing difficulties, and severe cases progress to acute respiratory distress syndrome requiring ventilation, and multi-organ failure, often resulting in death [3, 9, 10]. According to the World Health Organization, as of June 2022, there are over 533 million confirmed cases of SARS-CoV-2 reported and 6.3 million fatal cases of COVID-19. It is well established that the immune response plays a critical role in the pathogenesis of COVID-19. The timing and scale of the immune response to SARS-CoV-2 is a key factor influencing clinical outcome and pronounced immune dysregulation is observed during severe COVID-19 [11]. Drug treatments and concomitant disease that alter immune function are known to skew the response to SARS-CoV-2 and affect disease severity [12, 13].

Epidemiological studies have demonstrated that prior stroke is associated with poor COVID-19 outcome. Several meta-analyses have shown a history of cerebrovascular disease confers increased likelihood of hospital admission and mechanical ventilation in those with COVID-19 [14–18]. People with a history of cerebrovascular disease hospitalized with COVID-19 require an increased duration of intubation and display a lower rate of successful extubation than those without a history of cerebrovascular disease [19]. One pooled analysis of four clinical studies showed patients with prior cerebrovascular disease had a ~2.5-fold increased odds (95% confidence interval of 1.18–5.51) of experiencing severe COVID-19 [14]. While an association with severe disease is established, it is not yet clear if a history of stroke has a significant impact on COVID-19 mortality, with conflicting data in current publications [14, 20]. Further large-scale clinical studies are required to fully determine this risk. However, studies agree that a history of stroke increases risk of more severe COVID-19 disease. Additionally, it has been demonstrated that COVID-19 leads to increased risk of stroke [21, 22], and people who present to hospital with both COVID-19 and stroke display increased mortality compared with stroke patients without concurrent SARS-CoV-2 infection [23]. COVID-19 preceding stroke has been previously reviewed [24], and here we focus on the impact of stroke-induced immunomodulation on subsequent COVID-19 infection.

Prior to the COVID-19 pandemic, it was well established that stroke increases susceptibility to various infections. Infection is a common complication of stroke, occurring in up to one third of patients [25, 26]. Bacterial infections affecting mucosal surfaces such as the uro-genital and respiratory tracts are the most prevalent, and pneumonia, in particular, has been independently associated with increased short- and long-term morbidity and mortality [25, 27, 28]. Pneumonia is one of the most common forms of stroke-associated infection and correlates with increased length of hospital stay, a threefold increase in mortality, and poorer functional outcome in survivors [29–31]. In addition to risk factors including dysphagia and mechanical ventilation, enhanced vulnerability to infection is associated with a suppression of systemic immune function that follows stroke [32]. The suppression of systemic immunity is reported to begin within 12 h after stroke and can persist for weeks to months [33–36]; however, the temporal profile of post-stroke immune changes has not been comprehensively analysed. The majority of clinical studies have examined immune alterations at early time points following stroke when patients are in the hospital. However, systemic alterations to peripheral blood neutrophils, NK cells, B and T cell signalling and cytokine profiles have been described 90 days after stroke, and recently, it was shown that pro-inflammatory proteins persist for up to 7 years following stroke [35–37]. Understanding the immunological mechanisms mediating elevated susceptibility to infection and the persistence of immune alterations throughout stroke recovery is important for improving patient outcomes.

**Figure 1:**
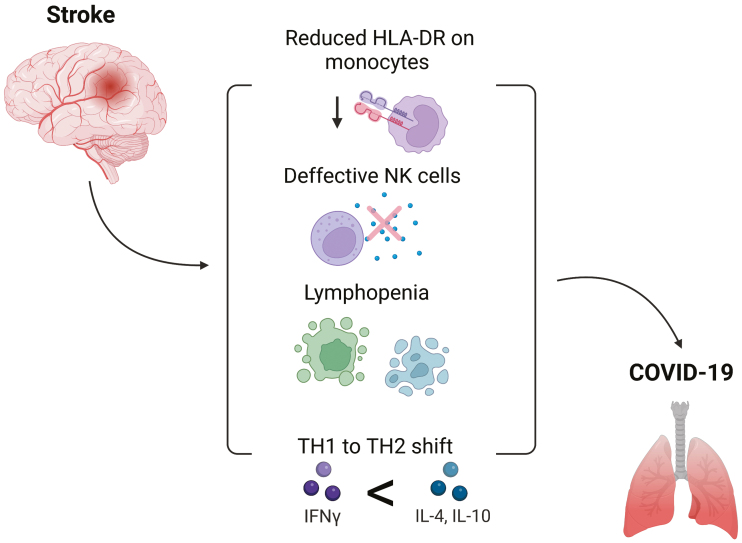
immunological features of acute stroke that might contribute to severe COVID-19. Changes to the immune system after stroke, include reduced HLA-DR expression on monocytes, defective NK cell functioning, lymphopenia, and a shift to a TH2-dominated response. These alterations may contribute to the increased risk of severe covid in patients with a history of stroke.

As with all pathogenic infections, the generation of a robust and timely immune response to SARS-CoV-2 is critical for the avoidance of severe disease. An effective anti-SARS-CoV-2 response relies on coordination by various components of the immune system, involving both the innate and adaptive arms. However, a number of the peripheral immune factors which contribute to the anti-viral response are suppressed following stroke. Severe atrophy of lymphoid organs and lymphopenia takes place, pro-inflammatory cytokines are downregulated, and multiple immune cell populations display functional deficiencies. This suppressed immune system might preclude or delay the generation of a potent anti-viral response, thus rendering people with a history of stroke more likely to develop severe disease. Additionally, a number of the immune alterations induced by stroke are also reported in severe COVID-19, including innate immune cell deficiencies, lymphopenia, and TH2-skewing (Fig. 1). Therefore, we postulate that the immunological profile of people recovering from stroke might compound many of the deleterious immune factors elicited by COVID-19.

Here, we review changes to systemic immunity following stroke and SARS-CoV-2 infection. We discuss the immunosuppression that follows stroke and highlight potential consequences for an effective anti-viral response. We also discuss the related immunological processes elicited by stroke and COVID-19, and posit that compounding effects might result in increased disease severity and poorer prognosis within this patient subgroup.

### Alterations to innate immune function in stroke and COVID-19

The immune system is broadly divided into innate and adaptive arms, both with important roles in protecting against and clearing infections. The innate immune system provides a first line of defence and plays a critical role in the anti-SARS-CoV-2 response. Intracellular viral infections are detected by the innate immune system via an array of pattern recognition receptors. RNA viruses, such as SARS-CoV-2, are detected by the endosomal RNA receptors, Toll-like receptor 3 (TLR3), TLR7, TLR8, and infected cells can detect the presence of the virus via the cytosolic RNA sensors, RIG-I and MDA5 [38]. Engagement of these RNA sensors results in signalling via IRF3/7 and the transcription of type I and III interferons (IFNs), in addition to various pro-inflammatory mediators and effector molecules. Type I IFNs are critical for restraining viral replication [39, 40] and loss of type I IFN functionality, either due to genetic variants or autoantibodies against IFNα, has been associated with severe COVID-19 [41–43]. Type I IFNs, in conjunction with other soluble mediators, facilitate recruitment and activation of various innate immune cells, including NK cells, macrophages, monocytes, and granulocytes. These populations contribute to viral clearance by recruiting leukocytes, destroying infected cells, presenting antigen, and repairing and restoring tissue homeostasis upon clearance. Following stroke, functional deficits in various innate immune cell populations have been identified. Below we discuss the innate immune response following SARS-CoV-2 and stroke and postulate that stroke might render decreased ability to restrain SARS-CoV-2 due to deficient innate immunity.

#### Monocyte macrophages

Monocytes and macrophages are innate mononuclear phagocytes that play critical roles host defence by recognizing pathogens and initiating inflammation. Along with dendritic cells (DCs), monocytes and macrophages make up the mononuclear phagocyte system. Macrophages are a heterogeneous population of tissue-resident mononuclear cells that maintain tissue homeostasis and respond to invading pathogens. Monocytes are bone-marrow-derived circulating phagocytes that are recruited to sites of inflammation where they take on inflammatory characteristics. During infection, monocytes and macrophages mount inflammatory responses by producing inflammatory molecules that facilitate recruitment of effector leukocyte populations, phagocytosing antigen, and acting as professional antigen-presenting cells. While critical for pathogen clearance, hyper-activated macrophages can also contribute to tissue damage. Following infection resolution, both monocytes and macrophages regulate tissue repair and the restoration of tissue homeostasis.

Various impairments to monocytes and macrophages are observed after stroke, including reduced abilities to produce pro-inflammatory cytokines and effectively present antigen, which might result in dampened anti-viral immune responses. Monocytes isolated from stroke patients or from rats following experimental stroke display reduced ability to produce the pro-inflammatory cytokines TNFα and IFNγ after stimulation with the bacterial endotoxin LPS [44–46]. These deficits were even more pronounced in stroke patients who developed stroke-associated infections, with further reductions in TNF-α and increased IL-10 [47]. Furthermore, recombinant tissue plasminogen activator (r-tPA), commonly used to restore blood flow to the brain after ischemic stroke, may exacerbate these effects as LPS-induced expression of TNF-α, IL-1β, IL-6, and CCL3 by bone marrow-derived macrophages *in vitro* was suppressed by both inactive and active forms of r-tPA [48]. Monocytes and granulocytes isolated from ischemic stroke patients up to 7 days following hospital admission also have a reduced ability to produce the enzymes myeloperoxidase and neutrophil elastase, which are crucial for their removal of pathogens [49]. Once again, r-tPA is thought to further compromise this ability (103). Monocytes/macrophages also have reduced ability to interact with cells of the adaptive immune system after stroke. Macrophages can present antigen on MHC Class II (HLA-DR in humans) to antigen-specific T cells, allowing the generation of specific adaptive immune responses. Reduced HLA-DR has been reported on monocytes derived from acute stroke patients in comparison to non-stroke controls [50, 51] and decreased expression of MHCII is observed on splenic macrophages following experimental stroke [52]. Macrophages also display reduced expression of various genes required for effective functioning following experimental stroke, including TLRs and co-stimulatory genes, suggesting that macrophages post-stroke might be less able to sense and respond to pathogens [52]. Isolated splenocytes, depleted of T cells, had a reduced capacity to co-stimulate exogenous T cells *in vitro* after experimental stroke which functionally demonstrates impairments in the ability of macrophages and DCs to activate the adaptive immune system [53]. Therefore, macrophages in people with a history of stroke may display deficiencies and be less able to contribute to an anti-viral response.

Recent studies have shown circulating monocyte subsets to be highly altered in SARS-CoV-2 infected patients. During SARS-CoV-2 infection, monocyte/macrophages are critical for an effective anti-viral response but can also contribute to prolonged and severe disease. The composition of the monocyte-macrophage populations is altered during SARS-CoV-2, with a particular expansion of CD14 + CD16 + inflammatory monocytes that are activated and able to secrete high concentrations of TNFαwhen stimulated *ex vivo* [54]. The predominant characteristic of monocytes during COVID-19 is loss of HLA-DR expression [55–57]. Importantly, reduction in HLA-DR expression correlates with more severe COVID-19 [58, 59]. The reduced HLA-DR-expression is associated with elevated expression of IL-10 and TGF-β, indicating a suppressive phenotype [60]. Monocytes from people with COVID-19 that lack HLA-DR expression are able to suppress T cell proliferation, functionally demonstrating their immunosuppressive capacities [61]. Together, these results highlight that an immunosuppressive monocyte population likely contributes to more severe disease. This raises the possibility that the macrophage-monocyte population in people with a history of stroke, which displays dampened effector capacity and reduced HLA-DR expression, might impede an effective anti-viral monocyte-macrophage response to SARS-CoV-2 and contribute to exacerbated disease.

#### Natural killer cells

Natural killer (NK) cells are innate-like lymphoid cells that quickly respond to viral infections by inducing the lysis of virus-infected cells [62]. NK cells show anti-SARS-CoV-2 activity [63], though the functionality of NK cells is impaired both in severe COVID-19 and post-stroke. NK cells are reduced in the circulation of COVID-19 patients and the extent of this reduction is associated with disease severity [64]. This may represent a loss of cells but could also reflect a recruitment of NK cells into the lungs of COVID-19 patients [54, 65]. NK cells from COVID-19 patients show reduced expression of IFNγ and TNFα [63, 64]. Highlighting the functional impairments, NK cells from people with COVID-19 co-cultured with virally-infected cells were less able to produce IFNγ and TNFα and reduce virus protein levels than th ose isolated from healthy individuals [63]. Similar functional deficits are observed in the acute phase following stroke, as NK cells isolated from stroke patients produce less IFNγ and perforin [66]. Recent studies have pinpointed the loss of NK cell effector function after stroke to reduced activity of the JAK/STAT3 pathway, since activation of this pathway specifically in NK cells after stroke successfully restored their function and reduced infection in an experimental animal model [67]. Alterations to NK cells in stroke and COVID-19 patients may be due to the high concentrations of IL-6 in the circulation, as NK cell numbers inversely correlate with IL-6 concentration and IL-6 has been shown to impair NK cell cytolytic functions *in vitro* [68]. Given that NK cells have a key role in identifying and killing virus-infected cells, and both COVID-19 and stroke can inhibit these functions, the combination of COVID-19 as a complication of stroke may further exacerbate deficits in NK cells that could increase disease severity.

### Alterations to systemic adaptive immunity in stroke and COVID-19

The adaptive arm of the immune system is equally as important for efficient defence against infection. Adaptive immunity is made up of CD4 and CD8 T cells and B cells, each of which interact with one another and innate immune cells, though each with a unique contribution to immunity. An effective adaptive immune response is critical in the response to SARS-CoV-2. However, following stroke, lymphocyte numbers are severely diminished and functional capacities of those that remain are reduced. Next, we will discuss the role of lymphocytes, with a focus on B and T cells, in the anti-SARS-CoV-2 response, and the impact of stroke on the adaptive compartment.

#### Lymphopenia

Stroke induces a rapid activation of the immune system with systemic pro-inflammatory cytokine production and recruitment of immune cells to the ischemic lesion. Simultaneously, a suppression of systemic immunity occurs, which may protect the brain from incurring further damage as a consequence of over-activation of inflammatory pathways, but consequently reduces defences against infection systemically [69, 70]. Extensive atrophy of secondary lymphoid organs, such as the spleen and thymus, has been described in both experimental animal models and in clinical studies [71–74] and reduced spleen size is associated with infection in stroke patients [75]. Lymphoid atrophy is primarily due to extensive apoptosis of lymphocytes within these organs, but migration of cells from peripheral immune organs to the brain or other sites of inflammation may additionally contribute to the reduced numbers [71, 76]. Systemic apoptosis of lymphocytes results in reduced B, T, and NK cells in the circulation following stroke [77]. This lymphopenia was first reported in experimental animal models and subsequently detected in patients as early as 6 hours after stroke and has been shown to persist out to at least 6 days [44, 74]. Lymphopenia is associated with an increased incidence of infection following stroke, with respiratory infection being the most common [25, 27, 77, 78].

The loss of lymphocytes is also a feature of severe COVID-19 [11, 79–81]. One study collected clinical data from over 1000 COVID-19 patients from various hospitals in China and showed lymphopenia was present in 83% of admitted patients [2]. The loss of lymphocytes is extensive, with the spleens and lymph nodes of those who succumbed to COVID-19 reported to harbour one third the number of T and B cells compared to individuals who died from non-COVID-19-related causes [82]. In particular, T cell numbers negatively correlate with patient survival during COVID-19 [81, 83–86]. The observed T cell cytopenia appears to be driven by increased Fas-mediated apoptosis, independent of direct viral infection, and is associated with more severe COVID-19 [87]. Extravasation into tissue and impaired proliferation could also contribute to observed lymphopenia. As stroke itself induces lymphopenia which increases infection risk, co-infection with SARS-CoV-2 may exacerbate this effect, further reducing a patient’s ability to fight secondary stroke-associated infections.

Lymphopenia is observed in combination with increased mobilization of granulocytes, including of monocytes, neutrophils, and eosinophils, in people with COVID-19 [88, 89]. This combination of lymphopenia and neutrophilia results in a high neutrophil to lymphocyte ratio (NLR), which has been associated with more severe COVID-19 [2, 79, 90, 91]. NLR is also a potential biomarker of prognosis in stroke, with an increased NLR associated with poorer outcome [92–94]. High NLR in people recovering from stroke with concurrent COVID-19 is therefore likely to indicate poor prognosis and increased disease severity and highlights the excessive risk of co-occurrence.

In addition to loss of lymphocytes following stroke, the remaining cells display functional deficiencies that likely further render elevated susceptibility to infection, which we will now discuss.

#### Modulation of the T cell and cytokine response

The ability to mount an efficient T cell-mediated immune response is critical for the clearance of SARS-Cov-2. T cells respond early during SARS-CoV-2 infection and play an important role in protection, but in severe disease, the T cells are aberrantly activated and T cell cytopenia occurs. One of the most important roles of T cells during anti-viral responses is the production of cytokines. A key feature of the immune response to SARS-CoV-2 is substantial production of pro-inflammatory cytokines, including IL-6, IL-1β, IL-8, TNFα, IL-2, and IFNγ [3, 11, 88, 95, 96]. Primarily produced by T cells and NK cells, but also macrophage monocytes, innate lymphoid cells, B cells, and others, inflammatory cytokines play critical roles in the anti-SARS-CoV-2 response. Alternately, aberrant production of cytokines is also known to contribute to severe disease. An array of studies have shown early increased concentrations of inflammatory cytokines, including of IL-1β, IL-4, and IL-6, are associated with more severe COVID-19 [3, 88, 97]. This highlights that a tightly regulated cytokine response is critical during SARS-CoV-2 infection, and indicates co-occurrences that skew this response will likely contribute to exacerbation of disease.

Following stroke, substantial skewing of the cytokine response is observed, with a shift from a TH1 (IFNγ) to TH2 (IL-4, IL-10) phenotype. This shift is characterized by reduced concentrations of circulating IFNγ and elevated IL-10 following stroke [98, 99]. Reduced production of IFNγ by lymphocytes persists for at least 3 months after stroke [99]. The anti-inflammatory TH2-dominated phenotype is thought to foster tissue repair and protect against additional brain injury, but contributes to infection vulnerability in patients [100, 101]. Increased circulating IL-10 and decreased IFNγ concentrations correlate with the development of infection in patients after stroke [36, 99, 102]. The predominance of TH2 over TH1 has been reported to persist up to 36 months after stroke in patients and may contribute to a long-term susceptibility to infection in addition to infectious complications experienced in the acute phase [103]. A systemic bias towards TH2 associated cytokines is also associated with more severe COVID-19. In particular, lower circulating IFNγis associated with more severe disease [104], while elevated IL-10 predicts poor COVD-19 outcomes [105, 106]. Therefore, stroke-induced skewing to a TH2-dominanted profile could again have the potential to further exacerbate COVID-19 severity.

In addition to a TH2 shift, there are also increases in inflammatory factors, including IL-6 and CRP, following stroke. IL-6 is one of the most studied biomarkers in stroke, as higher blood IL-6 concentration has been shown to positively correlate with severity and worse outcome [107–109] and the incidence of post-stroke infection [110, 111]. Acute phase proteins, such as CRP, are produced in the liver in response to pro-inflammatory cytokines such as IL-6 and are also increased in the acute phase of stroke [112]. CRP concentrations again have been associated with poor functional outcomes, increased mortality, and increased susceptibility to infection post-stroke [113–115]. CRP and IL-6 are also elevated in COVID-19 and are associated with worse prognosis [2, 116–118]. Since both stroke and COVID-19 lead to increases in circulating IL-6 and CRP, their combination could significantly exacerbate inflammatory mediator concentration and worsen overall patient outcome. CRP can activate the classical pathway of the complement cascade which is another important component of the innate immune response. Excessive activation of classical complement components is thought to contribute to cytokine storm and exaggerated pathology in severe COVID-19 disease, suggesting a potential mechanism by which elevated CRP levels after stroke may contribute to increased risk of severe disease [119].However, lectin pathway recognition molecules are able to bind to the SARS-CoV-2 spike and nucleocapsid proteins and activate the alternative arm of the complement cascade and is beneficial for infection clearance [120]. Stroke results in lower levels of circulating lectin pathway recognition subcomponents [121], which might restrict this arm of the complement cascade and result in increased susceptibility to infection establishment.

In addition to TH2 cytokines, regulatory T cells (Tregs) also suppress adaptive immune responses and are important for the prevention of uncontrolled inflammation. One subset of Tregs are identified by FOXP3^ + ^expression are increased in the circulation and spleen up to four days after experimental stroke [33, 122]. Immunomodulation by Tregs is thought to confer neuroprotective effects after stroke in patients and in experimental animal models by reducing harmful inflammatory responses and haemorrhagic transformation in the brain [123–125]. During SARS-CoV-2 infection, increased proportions of Tregs associate with worse clinical outcomes [126], again suggesting that the post-stroke and COVID-19 immune profile might overlap and contribute to more severe disease. Profiling immune cell subsets and circulating cytokines in acute stroke patients with COVID-19 in comparison to those with COVID-19 only will be key to understanding the specific risk of stroke patients of severe infection.

#### Altered humoral response

Experimental stroke causes a reduction in B cells in lymphoid tissues and the circulation, contributing to the generalized lymphopenia [71, 74, 127]. In addition to the splenic atrophy described above, the splenic architecture and follicle organization is dysregulated following experimental stroke [76]. The decreased B cell numbers coupled with a loss of follicle microarchitecture might inhibit the ability of people recovering from stroke to mount a robust humoral response, which is an important component of the anti-SARS-CoV-2 response. IgM and IgG antibodies specific to SARS-CoV-2 antigens increase following infection, with seroconversion occurring within weeks of infection onset [128]. Anti-SARS-CoV-2 antibodies can neutralize the virus and confer protection from infection both *in vitro* and *in vivo* [129–132]. However, more severe cases of COVID-19 are associated with elevated antibody titres [133, 134]. People who succumb to SARS-Cov-2 infection display an excessive humoral response, and yet also delayed production of neutralizing antibodies, compared to survivors [135]. This delay in seroconversion appears to put individuals at risk of severe disease and death. B cell-derived immunoglobulins, including IgG and IgM, are reduced acutely after stroke, and this dampened humoral response is associated with increased infection susceptibility [76, 136]. Stroke reduces the available pool of naïve B cells and additionally causes reduction in circulating antibody, and thus likely impairs a patient’s ability to generate a sufficient antibody response to SARS-CoV-2.

A reduction in IgM might be particularly relevant in the context of stroke, as following stroke, there is a disproportionate loss of IgM-producing marginal-zone (MZ) B cells. Following experimental stroke, MZ B cells are rapidly depleted and are associated with reductions in circulating IgM concentration, rendering increased susceptibility to spontaneous bacterial infections [76]. Circulating concentrations of IgM are reduced in people by 24 hours post-stroke and remain significantly lower than healthy individuals at 5–7 days following stroke [76]. IgM antibodies provide fast acting and protective immunity to microbial components, both bacterial and viral [137]. The loss of antibody neutralization capacity following SARS-CoV-2 infection is associated with the particular loss of anti-S IgM antibodies [138] and the specific depletion of IgM from the plasma of convalescent patients results in the most substantial decrease in virus neutralization of all isotypes [139]. The reduced pool of MZ B cells in people who have had strokes might result in a deficient anti-SARS-CoV-2 IgM antibody response and hindered ability to the effectively neutralize the virus.

### Hyperactivation of the immune system and severe COVID-19

We have detailed the immunosuppressive effects of stroke on the systemic immune system, and how these overlap with some of the immunological factors associated with severe COVID-19 disease may be responsible for the increased risk of severe infection in patients with a history of stroke. However, the development of severe COVID-19 disease and acute respiratory distress syndrome (ARDS) is also associated with a hyperactivation of the immune system with prolonged production of pro-inflammatory cytokines. The dysregulated immune response in severe COVID-19 disease includes a cytokine storm of pro-inflammatory mediators such as TNF-α, IL-1β, IL-6, and IL-8, resulting in hypotension, vascular leakage, multi-organ dysfunction, ARDS, and, in some cases, death [140]. Even in mild infection, this systemic inflammatory cytokine response is known to contribute to some of the neurological features of COVID-19 disease. In a humanized mouse model where a mild SARS-CoV-2 infection was limited to the lungs, microglial reactivity in the white matter tracts of the brain was detected and was associated with elevated cytokines and chemokines in the cerebrospinal fluid. Patients who experienced neurological symptoms of COVID-19 showed similar manifestations with impaired hippocampal neurogenesis, decreased oligodendrocytes, and myelin loss in subcortical white matter, suggesting that circulating inflammatory cytokines contribute to the neurological effects of COVID-19 disease [141]. This may be of increased significance in patients with a history of brain injury as respiratory infections are known to worsen cognitive impairment in patients on a trajectory towards dementia and could result in worsened neurological outcome in patients with a history of stroke [142].

## Summary

Stroke perturbs the systemic immune response, resulting in increased risk of infection. These alterations may compound and exacerbate immunopathology induced by SARS-CoV-2 infection. The extent to which immune deficits persist after stroke is currently uncertain. Stroke-induced deficits linked to increased susceptibility to infection have been mainly studied in the acute phase of stroke recovery. However, systemic alterations to peripheral blood neutrophils, B and T cell signalling and cytokine profiles have been described up to 90 days after stroke [35, 36], though it is unknown if the consequence of any such changes result in long-term infection susceptibility in either a clinical, or experimental, setting. Studies demonstrating increased risk of severe COVID-19 in patients with a history of cerebrovascular disease suggest that that stroke may have a lasting impact on how the immune system responds to infection and that it is not only in the acute phase of stroke recovery that this patient subgroup may have increased risk of poor outcome. Additionally, stroke-induced impairments of T cells, NK cells, and macrophages and shift in cytokine profile could be exacerbated by similar effects of COVID-19 on these cells, leading to a compounding effect and worsening overall disease severity. Studies investigating the immune response to COVID-19 in the context of stroke are necessary to understand the impact of these multiple immune stimuli on disease severity and their impact on both stroke and COVID-19 outcome.

Beyond the scope of this review but deserving of consideration is elucidating if stroke impairs SARS-CoV-2 protective immunity. Vaccination has proven to be an effective tool in reducing the incidence and severity of SARS-CoV-2 infection, however, we have little knowledge of how the extensive lymphopenia experienced after stroke impacts the adaptive immune system in the long-term. It is uncertain if people with a history of stroke generate efficient adaptive immune responses to new infection or vaccination. Of concern, IgM antibodies play an important role in protective immunity to SARS-CoV-2 [143, 144], and IgM titres are reduced following stroke [76], raising the possibility that people with a history of stroke might be less able to generate robust immunity following infection or vaccination. It is possible that vaccination against SARS-CoV-2, and other pathogens, might be less effective than in healthy individuals, and the number of doses required for effective protective immunity might be higher in people who have previously suffered from stroke.

Also worthy of consideration, in stroke we know that immune suppression is in part due to activation of stress pathways mediated via the sympathetic nervous system and the hypothalamic, pituatory, adrenal axis, resulting in increases in catecholamines, glucocorticoids and acetylcholine that can directly affect immune cells [74, 76, 145, 146]. Determining if similar pathways are activated in COVID-19 may give some insight into the upstream mechanisms underlying COVID-19 induced immune changes experienced in severe infections and provide some therapeutic targets to improve COVID-19 in the context of stroke.

Very few studies have been performed to understand if the immune deficits induced by stroke impair the anti-viral immune response in general, during either acute or chronic stroke recovery [147]. Respiratory viral infections such as influenza already have a high societal burden [148] and the impact of the current COVID-19 pandemic highlights the urgent need to understand these diseases within potentially immunocompromised patient groups. The established link between stroke and susceptibility to respiratory infection, and the complimentary effects of both stroke and COVID-19 on suppressing immune function, provide some insight into why stroke patients have an increased risk of severe COVID-19. Large-scale epidemiological analyses will be necessary to fully determine the extent to which stroke increases the risk of developing severe COVID-19 both in the chronic and acute recovery phases, and experimental and clinical studies are necessary to determine the immunological mechanisms that underpin the increased risk of severe COVID-19 disease after stroke. Given the available evidence, stroke patients could be considered an “at risk” group and prioritized for vaccination boosters and any other future strategies to reduce infections in vulnerable populations. However until we understand the long-term effects of stroke on the immune system and how these contribute to sustained risk of more severe infection during recovery, we cannot discern whether alternative or adjunct treatment regimen are required when treating COVID-19 in patients with a history of stroke.

## Data Availability

No new data was generated for the publication of this manuscript.

## Permission to Reproduce

This manuscript is published under a CC-BY licence allowing use, reproduction, dissemination, or display of the article in any way, including for commercial purposes, so long as the authors are credited for the original creation.
